# Characterization of SARS-CoV-2 Variants in Military and Civilian Personnel of an Air Force Airport during Three Pandemic Waves in Italy

**DOI:** 10.3390/microorganisms11112711

**Published:** 2023-11-05

**Authors:** Michele Equestre, Cinzia Marcantonio, Nadia Marascio, Federica Centofanti, Antonio Martina, Matteo Simeoni, Elisabetta Suffredini, Giuseppina La Rosa, Giusy Bonanno Ferraro, Pamela Mancini, Carolina Veneri, Giovanni Matera, Angela Quirino, Angela Costantino, Stefania Taffon, Elena Tritarelli, Carmelo Campanella, Giulio Pisani, Roberto Nisini, Enea Spada, Paola Verde, Anna Rita Ciccaglione, Roberto Bruni

**Affiliations:** 1 Department of Neurosciences, Istituto Superiore di Sanità, 00161 Rome, Italy; michele.equestre@iss.it; 2 Department of Infectious Diseases, Istituto Superiore di Sanità, 00161 Rome, Italy; cinzia.marcantonio@iss.it (C.M.); centofantifederica@outlook.it (F.C.); angela.costantino@iss.it (A.C.); stefania.taffon@iss.it (S.T.); elena.tritarelli@iss.it (E.T.); roberto.nisini@iss.it (R.N.); enea.spada@iss.it (E.S.); annarita.ciccaglione@iss.it (A.R.C.); roberto.bruni@iss.it (R.B.); 3 Clinical Microbiology Unit, Department of Health Sciences, “Magna Grecia” University, 88100 Catanzaro, Italy; mmatera@unicz.it (G.M.); quirino@unicz.it (A.Q.); 4 Center for Immunobiologicals Research and Evaluation, Istituto Superiore di Sanità, 00161 Rome, Italy; antonio.martina@iss.it (A.M.); matteo.simeoni@iss.it (M.S.); giulio.pisani@iss.it (G.P.); 5 Department of Food Safety, Nutrition and Veterinary Public Health, Istituto Superiore di Sanità, 00161 Rome, Italy; elisabetta.suffredini@iss.it; 6 Department of Environment and Health, Istituto Superiore di Sanità, 00161 Rome, Italy; giuseppina.larosa@iss.it (G.L.R.); giusy.bonannoferraro@iss.it (G.B.F.); pamela.mancini@iss.it (P.M.); carolina.veneri@iss.it (C.V.); 7 Clinical Analysis and Molecular Biology Laboratory Rome, Institute of Aerospace Medicine, 00185 Rome, Italy; carmelocampanella21@gmail.com; 8 Aerospace Medicine Department, Aerospace Test Division, Militay Airport Mario De Bernardi, Pratica di Mare, 00040 Rome, Italy; paolaverde26@gmail.com

**Keywords:** SARS-CoV-2 variant, 3CLpro, RdRp, Plpro, spike, nucleocapsid, mutations, Sanger, Next-Generation Sequencing

## Abstract

We investigated SARS-CoV-2 variants circulating, from November 2020 to March 2022, among military and civilian personnel at an Air Force airport in Italy in order to classify viral isolates in a potential hotspot for virus spread. Positive samples were subjected to Next-Generation Sequencing (NGS) of the whole viral genome and Sanger sequencing of the spike coding region. Phylogenetic analysis classified viral isolates and traced their evolutionary relationships. Clusters were identified using 70% cut-off. Sequencing methods yielded comparable results in terms of variant classification. In 2020 and 2021, we identified several variants, including B.1.258 (4/67), B.1.177 (9/67), Alpha (B.1.1.7, 9/67), Gamma (P.1.1, 4/67), and Delta (4/67). In 2022, only Omicron and its sub-lineage variants were observed (37/67). SARS-CoV-2 isolates were screened to detect naturally occurring resistance in genomic regions, the target of new therapies, comparing them to the Wuhan Hu-1 reference strain. Interestingly, 2/30 non-Omicron isolates carried the G15S 3CLpro substitution responsible for reduced susceptibility to protease inhibitors. On the other hand, Omicron isolates carried unusual substitutions A1803V, D1809N, and A949T on PLpro, and the D216N on 3CLpro. Finally, the P323L substitution on RdRp coding regions was not associated with the mutational pattern related to polymerase inhibitor resistance. This study highlights the importance of continuous genomic surveillance to monitor SARS-CoV-2 evolution in the general population, as well as in restricted communities.

## 1. Introduction

Italy was one of the worst afflicted countries during the Severe Acute Respiratory Syndrome Coronavirus 2 (SARS-CoV-2) pandemic, with 101,739 infected cases and more than 10,000 people deceased by March 2020. Due to rapid decision making, on 27 December 2020, the Italian Medicines Agency approved the BioNTech/Pfizer mRNA BNT162b2 Comirnaty vaccine and started the first stage of the vaccination campaign, initially targeting healthcare workers [1]. By 1 May 2021, over 20,000,000 single doses of vaccine had been administered. By March 2023, 40,478,062 people had been administered with a booster dose of vaccine [2]. To control and monitor the SARS-CoV-2 pandemic, the World Health Organization (WHO) and the European Centre for Disease Prevention and Control (ECDC) recommend Whole Genome Sequencing (WGS), or complete/partial spike (S) gene sequencing to detect new variants with variable severity and infectivity, as well as immune escape potential [3,4,5]. During the pandemic, several variants were identified and subsequently designated by WHO as Variants Being Monitored (VBMs), Variants of Interest (VOIs) or of Concern (VOCs), characterized by greater transmissibility, virulence, and resistance, and finally, Variants of High Consequence (VOHCs), which are highly dangerous and epidemic [5,6].

The first VOCs to emerge in Italy were the Alpha (B.1.1.7) VOC, first identified in the UK (September 2020) and around 30–40% more transmissible than the original wild-type, the Beta (B.1.351) VOC, first documented in South Africa (May 2020), and the Gamma (P1) VOC, first identified in Manaus, Brazil (November 2020), 1.7–2.4 times more transmissible than wild-type [7]. From June 2020, two novel SARS-CoV-2 variants were detected in the Italian territory, the B.1.177 (20A.EU1) and the B.1.258 (20A) variants [7]. The latter represented an example of a nationally prevailing variant not commonly detected in other countries at that time, but later spread globally, likely due to the acquisition of beneficial mutations including ∆H69/V70 deletions, N439K, and D614G, all concentrated in the S1 subunit, specifically at the start of the N-terminal domain (NTD) and in the receptor-binding domain (RBD) [8]. The detection of S gene mutations is of particular importance in monitoring emerging variants able to enhance virus transmission, reinfection, and vaccine escape [9]. Additionally, recent data highlight the high mutational rate of the nucleocapsid (N) gene and its implication on pathogenesis and on diagnosis failure by commercial assays [10,11].

Based on genomic surveillance, surveys conducted across the whole Italian territory during the first month of 2021 highlighted the Alpha variant as dominant, followed by a substantial proportion of cases of the Gamma variant, almost exclusively in central Italy [12]. These two variants decreased during the summer of 2021, when the number of reported cases caused by the Delta variant (B.1.617.2) exceeded those caused by the Alpha. More than 80% of cases in all age groups were caused by the Delta variant, with the highest percentage in subjects aged between 10 and 29 years [13]. The Delta variant was characterized by higher transmissibility, viral RNA loads, and infectivity than the Alpha in both vaccinated and unvaccinated individuals [14]. On 27 November 2021, the Omicron variant was identified for the first time in Italy. Nine cases were reported in four regions, and all patients were either asymptomatic or with mild or cured symptoms [13]. The Omicron variant (BA) rapidly spread across the country, and its prevalence increased from 1.0% to 65.9% in only three weeks, reaching 80% in the first week of January, when it became predominant [15]. Recently, the XBB.1.5 (also known as Kraken), plus F456L substitution VOI and the EG.5 (also known as Eris), and BA.2.86 (also known as Pirola), VBMs were detected globally [16]. These new SARS-CoV-2 variants are potentially able to cause breakthrough infections in people with previous immunity (infected or vaccinated) [16]. Even if hospitalization rates and severe illness have not yet increased, the COVID-19 vaccine targeting the XBB.1.5 VOC is ongoing [17]. It is expected to be effective against the new variants identified during the current international epidemiological surveillance [17]. The large number of published papers and available sequences were, and still are, very useful for monitoring SARS-CoV-2 spread direction, noting each new mutation and its potential role on transmissibility and disease severity [9,18,19,20].

Since 2021, some antivirals are available for treating viral infection in combination or not with monoclonal antibodies (mAbs), most of which are ineffective due to immune escape [9,21]. New antivirals target viral enzymes involved in replication, such as 3-Chymotrypsin-like protease (3CLpro), RNA-dependent RNA polymerase (RdRp), and the papain-like protease (PLpro) [22]. Naturally occurring resistance mutations, already fixed in the viral population, or selected resistance under drug pressure could be responsible for treatment failure [23]. In this regard, SARS-CoV-2 molecular characterization, used to monitor antiviral resistance of different variants, can help prioritize healthcare resources and optimize patient management strategies. The most at-risk patients treated by a combination therapy were not hospitalized and had no adverse events [22,24]. Furthermore, this treatment could reduce selection of SARS-CoV-2 resistant isolates [24].

Since the beginning of the pandemic, the Italian Government, in order to cope with the transmission of COVID-19, enacted a series of control measures that limited interaction between people, such as social distancing, closure of many commercial activities, and implementation of home working. Simultaneously, public health services were improved to control any outbreak occurring in restricted areas by contact tracing [25]. In the globalized era, air transport and passenger mobility are essential for economic development, but they also increase risks associated with the transmission of COVID-19 disease in airport areas due to the provenance of travelers from different countries and latitudes, making early identification and isolation of asymptomatic carriers necessary to avoid possible outbreaks [25]. Similarly, Air Force personnel deployed to military bases abroad may be a hotspot for virus transmission, due to interaction with both military and civilian personnel from different nations. The screening program and molecular epidemiology studies can help to monitor and limit the spread of the virus within these communities by identifying potential outbreaks and implementing appropriate control measures [3,4,5,26].

In this scenario, from November 2020 to March 2022, a COVID-19 screening program and surveillance testing was conducted among the military and civilian personnel of the “Mario De Bernardi” military airport, within the framework of the Scientific Collaboration Protocol signed by the Experimental Flight Center—Italian Air Force Logistic Command—and the Istituto Superiore di Sanità, with the aim of reducing viral transmission among personnel attending the military airport base [27].

Herein, we analyzed SARS-CoV-2 positive nasopharyngeal swabs using Next-Generation Sequencing and Sanger methods to characterize variants spread in the restricted community of the military airport during three pandemic waves in Italy. We focused attention on known and unusual mutations in genomic regions, the target for new therapies (3CLpro, RdRp, PLpro), and N and S coding regions, both important genomic hotspots, also involved in routine diagnosis. In addition, as a secondary objective, we evaluated the performance of partial sequencing of the spike coding region in identifying different variants accurately by comparison with results from whole genome sequencing.

## 2. Materials and Methods

### 2.1. Ethical Statement

The study protocol was approved and signed by the Scientific Collaboration Partners (i.e., Experimental Flight Center, Italian Air Force Logistic Command and Istituto Superiore di Sanità) on 30 November 2020. Personal data were collected and processed in compliance with EU and Italian legislation [28,29,30]. Written informed consent was obtained from all participants or their legal representative. All activities were carried out as public health activities to improve the tracing of infected individuals and contacts with the aim of reducing virus transmission among personnel attending the military airport base.

### 2.2. Study Design

**Figure 1:**
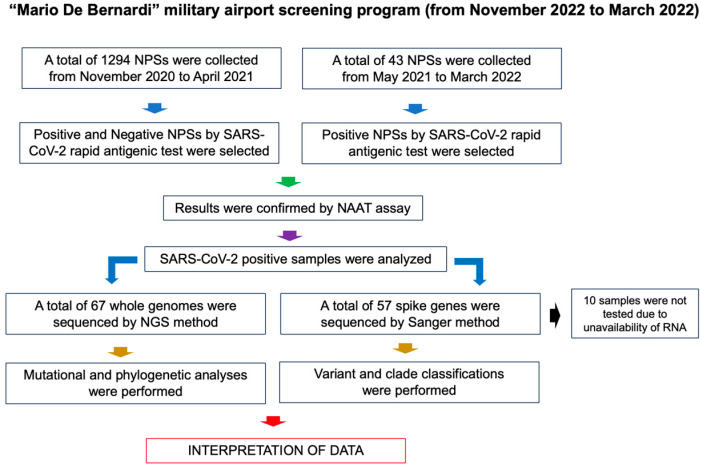
Study design. NPSs, nasopharyngeal swabs; NAAT, Nucleic Acid Amplification; NGS, Next-Generation Sequencing.

From November 2020 to March 2022, the military and civilian personnel of the “Mario De Bernardi” military airport, located in the metropolitan area of Rome, underwent a screening program to control workplace transmission of SARS-CoV-2 infection. According to the national legislation [31,32,33], testing was performed locally by a first-line rapid antigen test, then positive and negative nasopharyngeal swabs (NPSs) were sent to the Istituto Superiore di Sanità (ISS) for confirmation of results by Nucleic Acid Amplification (NAAT) (overall, 1294 samples were included) [27]. From May 2021 to March 2022, only rapid antigen test positive samples were sent to ISS for confirmation by NAAT (43 samples in total). The subjects with confirmed SARS-CoV-2 positive test by gold standard real-time reverse transcription-polymerase chain reaction (RT-PCR) were included in the study. Positive samples were subjected to Next-Generation Sequencing (NGS) of the whole viral genome and Sanger sequencing of the spike coding region. Newly generated sequences were analyzed by molecular analyses. The study design is reported in Figure 1.

The study was part of the public health response to control any outbreak occurring in the military airport as soon as possible (as reported in the Scientific Collaboration Protocol, see above). All participants were asked to complete a questionnaire including demographic data, symptoms (if any), and potential exposure to infection (previous COVID-19, contact with proven COVID-19 cases, or contact with persons who tested positive for SARS-CoV-2 by molecular or antigenic tests). Subsequently, the data collected for the screening purpose were linked to the SARS-CoV-2 variants resulting from sequencing to better characterize the positive cohort.

### 2.3. Diagnostic Reverse Transcription (RT) Real-Time PCR

The confirmation of rapid antigen test results was based on a RT-qPCR assay (RealStar^®^ SARS-CoV-2 RT-PCR Kit 1.0, Altona Diagnostics, Hamburg, Germany) for the detection of envelope (E) and spike (S) genes. According to manufacturer’s procedures, 10 μl of RNA was used for a one step reaction to synthetize cDNA and amplify viral genome. The cDNA was used as a template with the following PCR conditions: 95 °C for 2 min, 45 cycles of 95 °C for 15 s, 55 °C for 45 s, and 72 °C for 15 s. RNA was extracted using the QIAamp^®^ MinElute^®^ Virus Spin Kit (QIAGEN, Hilden, Germany). Nucleic acid purification was performed using the QIAcube instrument (QIAGEN Biotechnology & Life Science). The purified RNA was amplified by RT-qPCR technology on the Rotor-Gene^®^ instrument (QIAGEN, Hilden, Germany) [27]. 

### 2.4. SARS-CoV-2 NGS Sequencing

Sixty-seven SARS-CoV-2 sequences were collected from positive individuals by real-time RT-PCR assay. Viral nucleic acids were extracted from swab samples using QIAamp MinElute Virus Spin Kit (Qiagen, GmbH, Hilden, Germany). To generate complete genome sequences, a targeted amplicon-based approach was utilized, employing the Illumina COVIDSeq Assay (96 samples). This assay incorporates a pool of primers whose design was based on the well-established and publicly accessible ARTIC consortium multiplex PCR protocol, specifically developed for the detection and characterization of SARS-CoV-2 RNA [34]. The Illumina COVIDSeq Assay involved the multiplex amplification of overlapping PCR products, preceded by a reverse transcription step to convert SARS-CoV-2 RNA into cDNA. The resulting amplified fragments were used to construct a library for Illumina deep sequencing, utilizing the Nextera XT DNA Library Preparation and Index kits (Illumina, San Diego, CA, USA). Sequencing was performed with a pair end approach on the Illumina MiSeq platform using the MiSeq Reagent Kit v3 [35].

The obtained sequence data were subjected to analysis using the Illumina^®^ DRAGEN COVID Lineage App [35]. Briefly, following base calling and quality scoring, the resulting sequence reads underwent adapter removal and trimming of ends with low-quality scores. The remaining high-quality reads were then mapped and aligned to the Wuhan-hu-1 reference sequence (Accession Number MN908947.3). Sequence variants were identified using the DRAGEN Somatic caller and the high confident ones incorporated into the reference genome to generate a consensus genome for the sequences based on the selected reference [35]. The first lineage/clade analysis on the consensus genomes was run using Pangolin [36] and/or NextClade [37].

### 2.5. SARS-CoV-2 Sanger Sequencing

Overall, of the 67 samples that underwent whole genome sequencing, 57 were also subjected to long PCR and Sanger sequencing for the purpose of comparison [38]. The remaining 10 samples could not be tested using PCR due to the unavailability of RNA.

The long-nested RT-PCR assay amplifies approximately 1600 bps spanning the region coding for amino acids 58 to 573 of the spike protein, allowing for the detection of multiple nucleotide changes resulting in key spike protein mutations distinctive of circulating SARS-CoV-2 variants. This assay has been successfully used for the screening of both clinical and environmental samples [39,40]. After amplification, the products were run on a QIAxcel Connect System, a capillary electrophoresis instrument for easy and cost-effective fragment analysis (Qiagen). The PCR products of the expected size were purified using a Montage PCRm96 Microwell Filter Plate (Millipore, Billerica, MA, USA) and sequenced by Sanger sequencing through an external service (Bio-Fab Research, Rome, Italy) [39,40]. Consensus sequences were generated using Molecular Evolutionary Genetics Analysis (MEGA X) software version 10 [41,42]. CoVsurver, enabled on the GISAID website, was used to analyze SARS-CoV-2 mutations by comparison with the reference strain hCov-19/Wuhan/WIV04/2019 [43].

### 2.6. Classification and Mutational Analysis

SARS-CoV-2 viral isolates were classified by Pangolin COVID-19 Lineage Assigner v.4.0.6 [36]. The following genomic regions were analyzed: ORF1ab (21,289 nucleotides, nt), S (3821 nt), ORF3a (827 nt), E (Envelope, 227 nt), M (Membrane, 668 nt), ORF6a (185 nt), ORF7a (365 nt), ORF7b (131 nt), ORF8 (365 nt), N (1259 nt), and ORF10 (116 nt). Amino acid changes of the whole genome were submitted to CoVsurver, Stanford Coronavirus Resistance Database (CoV-RDB), and Nextclade web servers [18,37,41]. The amino acid (aa) changes were further confirmed by comparing newly identified isolates with the SARS-CoV-2 Wuhan-Hu-1 reference sequence (accession NC_045512) downloaded from the GenBank^®^ database [44]. Mutations were noted on the following predicted proteins: non-structural protein 1 (nsp1, 180 aa), nsp2 (638 aa), nsp3 (PLpro, 1944 aa), nsp4 (499 aa), nsp5 (3CLpro, 605 aa), nsp6 (289 aa), nsp7 (82 aa), nsp8 (197 aa), nsp9 (112 aa), nsp10 (138 aa), nsp12 (RdRp, 931 aa), nsp13 (600 aa), nsp14 (526 aa), nsp15 (345 aa), nsp16 (297 aa), S (1273 aa) NS3a (275 aa), E (75 aa), M (222 aa), NS6 (61 aa), NS7a (121 aa), NS7b (43 aa), NS8 (121 aa), N (419 aa), NS10 (38 aa).

### 2.7. Phylogenetic Analysis

The 67 final assembled whole genome sequences were aligned by ClustalW in BioEdit alongside 17 reference sequences (including the Wuhan-hu-1 reference and representatives of the major SARS-CoV-2 strains/VOCs) [45,46]. Then, the aligned sequences were checked and, wherever needed, manually edited to optimize alignment accuracy. The best substitution model for the dataset under analysis was evaluated by the Models tool available in MEGA software version 10 [41,42]. A phylogenetic tree was constructed using the Maximum Likelihood approach implemented in MEGA, with the estimated best substitution model (GTR + G + I). Statistical reliability of the phylogenetic tree was assessed by bootstrap analysis (1000 replicates); a bootstrap value > 70 was considered significant [47].

## 3. Results

### 3.1. Demographic Characteristics of the Cohort

Overall, 67 SARS-CoV-2 sequences were collected from individuals who were positive by real-time RT-PCR assay. Thirty sequences were classified as non-Omicron variants and thirty-seven as Omicron or its sub-lineages. Table 1 reports information about these two groups of the studied cohort, including referred symptoms.

The median age of the 30 non-Omicron subjects was 38.1 years old (range 13–55) and there was a greater proportion of males (75.9%) than females (24.1%). The reported COVID-19 symptoms at the time of diagnosis were typical in 17 subjects, while atypical in 10 individuals (70% males). Interestingly, chronic co-morbidities were reported in three male individuals. Previous SARS-CoV-2 positive swab or COVID-19 diagnosis was reported by 12 subjects.

Among the 37 individuals infected by Omicron and its sub-lineages, the median age was 43.4 years (range 15–74) and the percentage of females was 37.8%. All individuals presented specific COVID-19 symptoms except six subjects; chronic diseases were reported in 15 cases. Most of the subjects had been in contact with SARS-CoV-2 swab-positive individuals or had contact with COVID-19 sick people.

### 3.2. Classification and Timing of Detected Variants

**Figure 2:**
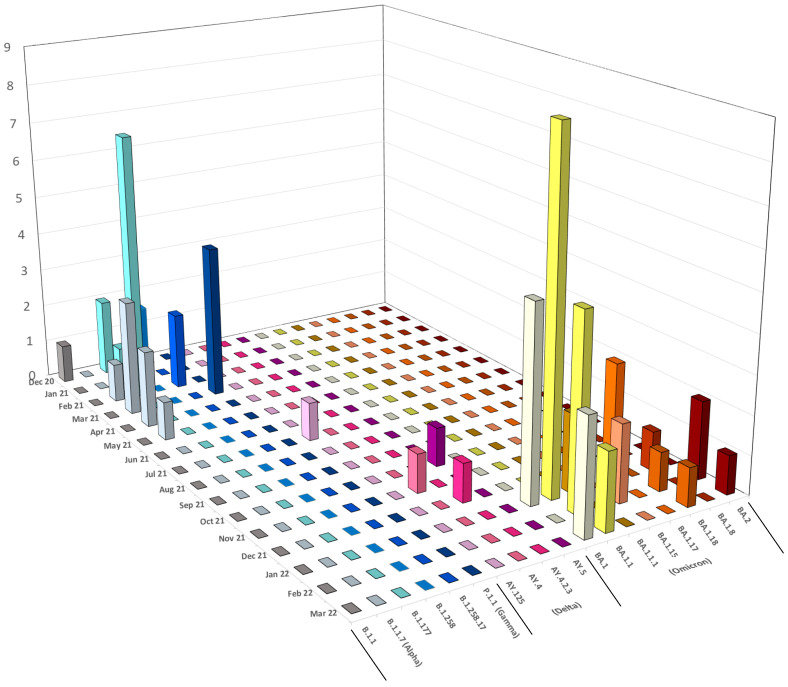
SARS-CoV-2 variants dynamic distribution between December 2020 and March 2022 in the military airport. The colors identify the different SARS-CoV-2 variants during the time span.

In Italy, three pandemic waves were observed between December 2020 and March 2022. In the same time span, we sequenced 67 SARS-CoV-2 genomes during the screening program in the “Mario De Bernardi” military airport. Since the end of December 2020, several variants became dominant, leading up to the beginning of the Omicron era; this latter variant became continuously dominant in the first months of 2022 (Figure 2). In our cohort, we identified the B.1.258 and sub-lineages (4/67), B.1.177 variant (9/67), Alpha (B.1.1.7) VOC (8/67), Gamma (P.1.1) VOC (4/67), Delta VOC and AY.125, AY.4, AY.4.2.3, AY.5 sub-lineages (4/67), finally Omicron VOC and BA.1.1, BA.1.1.1, BA.1.15, BA.1.17, BA.1.18, BA.1.8, BA.2 sub-lineages (37/67).

### 3.3. Whole Genome Analysis

**Figure 3:**
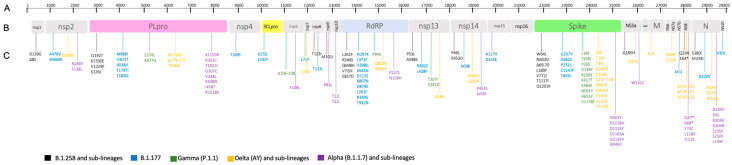
(A) SARS-CoV-2 whole genome length. (B) Polyprotein subdivided in structural and non-structural proteins. (C) Amino acid substitutions of non-Omicron variants and their sub-lineages were reported for each variant with a different color according to the viral isolates (little square). The * symbol identified the stop codon.

**Figure 4:**
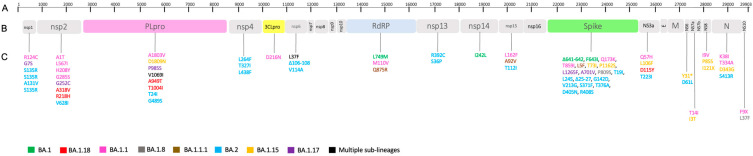
(A) SARS-CoV-2 whole genome length. (B) Polyprotein subdivided in structural and non-structural proteins. (C) Amino acid substitutions of Omicron VOC and its sub-lineages were reported for each variant with a different color according to the viral isolates (little square). The * symbol identified the stop codon.

Genomic analysis of 67 isolates showed a high number of amino acid (aa) changes in whole genomes (Supplementary Tables S1 and S2). The substitutions with low prevalence (unusual) among newly generated sequences, detected on the whole genome of non-Omicron (Figure 3) and Omicron (Figure 4) variants were noted.

#### 3.3.1. PLpro Protein

The PLpro showed the highest number of mutations considering the whole ORF1 region. We found a total of 22 unusual aa substitutions among the 30 non-Omicron isolates, most of which related to the Alpha. A total of two B.1.258 isolates displayed the K1330E, two B.1.177 isolates showed the same three mutations (A534V, T1797I and T1830I), three Gamma VOC carried the substitution S370L, plusK977Q on two isolates, two Delta VOC displayed the A1711V substitution, and three Alpha VOC carried the G307C, V348L, and K408N mutations. Unique mutations detected on PLpro protein of non-Omicron isolates were: I1192T, V1229F, S126L, M988I, I1672T, ∆174-177, T936N, K1155R, R352C, T1022I, I458T, and R1518K (Supplementary Table S1). Among Omicron isolates, we found eight unusual aa substitutions. In particular, the A1803V, D1809N, and A949T substitutions were present in one out of 37 isolates, respectively. All three isolates belonging to BA.1.17 sub-lineage and one BA.1.18 isolate showed the V1069I aa change, while all BA.2 isolates carried the double mutation T24I and G489S (Supplementary Table S2).

#### 3.3.2. 3CLpro Protein

Two major mutations characterize this protease in non-Omicron VOC isolates: G15S and L232F, both belonging to B.1.177 (Supplementary Table S1). An Omicron VOC isolate related to the BA.1.1 sub-lineage carried the amino acidic substitution D216N, which is very uncommon worldwide (Supplementary Table S2).

#### 3.3.3. RdRp Protein

Eighteen unusual mutations were observed in the RdRp protein of non-Omicron VOC isolates. Seven (N297K, L371F, V398IL, K641N, D717E, K807N, and D879E) were carried by the same B.1.177 isolate. The L261F and R349S substitutions were both shown by B.1.177 and B.1.258 isolates, the latter also carrying Q698H and E857D mutations. Other unusual mutations were found in the genomic region of different isolates: V720I (1/30), T912N (1/30), P94S (3/30), Q822H (1/30), D824Y (1/30), P227L (1/30), and N158H (2/30) (Supplementary Table S1). Similarly, genomic analysis of Omicron VOC isolates showed three uncommon aa changes in BA.1, BA.1.1, and BA.1.1.1 sub-lineages: L749M (1/37), M110V (1/37), and Q875R (2/37), respectively (Supplementary Table S2).

#### 3.3.4. Spike Protein

In the S glycoprotein of non-Omicron isolates, we found 12 unusual mutations, which were related to both B.1.258 and B.1.177 variants. The N603D, G257V, and T859I were detected only in single isolates. Six unusual mutations were observed in Alpha isolates, including D1153Y (1/30), G1124V (1/30), G946V (1/30), and the unique D1165A substitution. Furthermore, a total of 21 unusual mutations were observed in Gamma and Delta isolates, among which L5F, Y145H, and V1104L (each one present in 1/30) and G142D (carried by 2/20 isolates) (Supplementary Table S1).

A higher number of aa substitutions was observed in Omicron isolates, including 19 unusual mutations such as Q173K (1/37), T859I (1/37), L1265F (2/37), A701V (1/37), P809S (1/37), and L5F (1/37) already present in the Delta VOC. One isolate (BA.1) carried the ∆641–642 deletion and F643I substitution. One isolate (BA.1.15) showed the T73I plus P1162S change, while the three isolates belonging to BA.2 sub-lineage carried the T19I, L24S, Δ25–27, G142D, V213G, S371F, T376A, D405N, and R408S mutations (Supplementary Table S2).

#### 3.3.5. Nucleocapsid Protein

The analysis of the N protein of non-Omicron isolates showed the presence of 12 unusual mutations among the non-Omicron VOC isolates, of which the S250F substitution deserves special attention. Eleven Alpha isolates carried the double amino acid change R203K and G204R plus S235F in 6/9 isolates. One Delta isolate showed both the S327L and W330L substitutions. Other observed mutations were S180I and M234I, detected in two and one B.1.258 isolates, respectively. The A220V was carried by seven B.1.177 variant isolates and by one Alpha. The R32H was detected in one Delta. The L139F (2/30) and D3L (8/30) were detected in Alpha isolates (Supplementary Table S1).

Among non-Omicron VOCs, two subjects who were SARS-CoV-2 positive by real-time RT-PCR (median Ct: 24) had tested negative by the first-line rapid antigen test used for the screening, targeting the N protein: one of them (AM-0607 isolate, belonging to B.1.258) displayed the S180I substitution, while the other (AM-0143 isolate, belonging to Alpha VOC) carried the A220V substitution.

Among Omicron isolates, the BA.1.1 sub-lineage showed uncommon mutations, such as K38I and T334A. Both BA.1.15 isolates carried the amino acid substitution D343G, while all BA.2 isolates showed the mutation S413R (Supplementary Table S2). In five cases, the SARS-CoV-2 isolates AM-0074, AM-0080, AM-0258, AM-0953, and AM-1185 were detected by real-time RT-PCR but not by rapid antigen testing. The median Ct values were 32, 31, 29, 15, and 23, respectively.

### 3.4. Phylogenetic and Clustering Analyses

**Figure 5:**
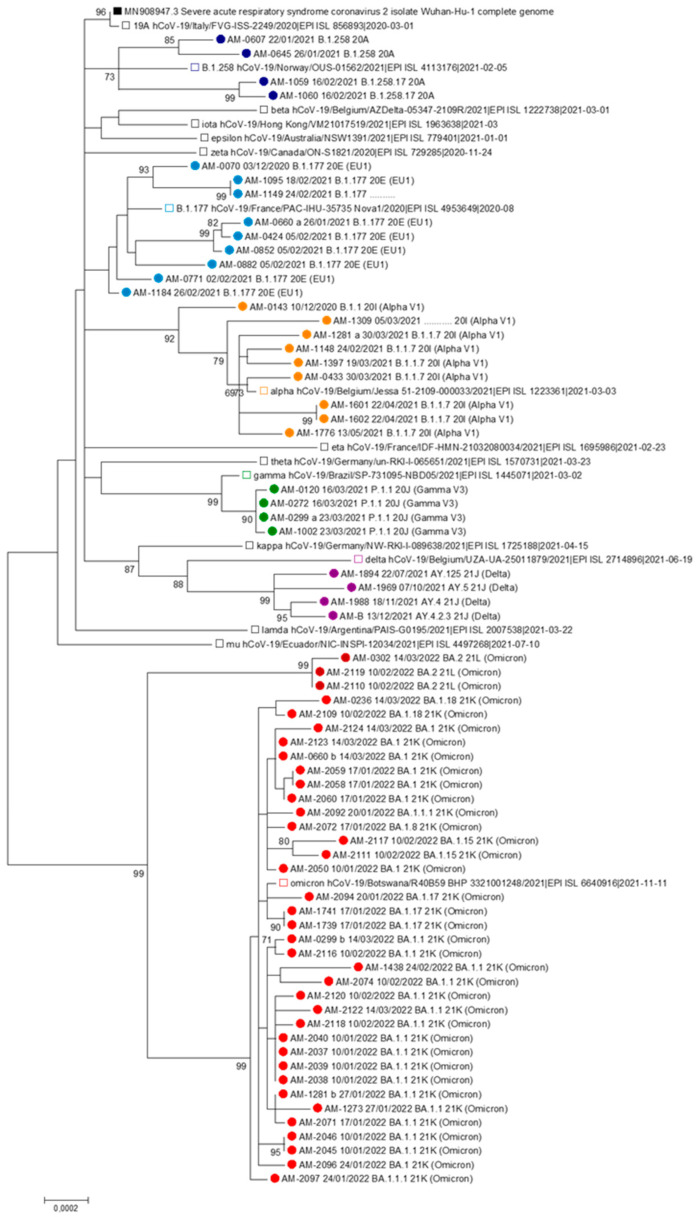
ML phylogenetic tree was estimated using 17 reference sequences and 67 SARS-CoV-2 newly generated sequences. The reliability of the phylogenetic clustering was evaluated using bootstrap analysis with 1000 replicates. The bootstrap support values > 70% are shown. The scale bar at the bottom of the figure represents genetic distance. The colors identify the several clusters of SARS-CoV-2 variants. The circles show the newly generated sequences, while the squares the reference sequences.

The phylogenetic relationship of SARS-CoV-2 circulating strains within the military airport was performed by molecular analysis of the sequences aligned to Wuhan Hu-1 and to several VOC reference sequences, to designate clades and lineages (Figure 5).

In our samples, we identified seven clades according to specific time span: clade 20E (12.1%) between December 2020–February 2021; clade 20I (13.6%) December 2020–March 2021; clade 20A (6.1%) between January–February 2021; clade 20J (6.1%) in March 2021; clade 21J (6.1%) between July–December 2021; clade 21K (51.5%) between January–March 2022; finally, clade 21L (4.5%) between January–February 2022. One viral strain, classified as B.1.177 by Pango lineage, was unclassified by Nextstrain nomenclature. The clades 21K and 21L, related to Omicron VOC, represented most of the sequences (56.0%) included in the phylogenetic tree, according to their high prevalence in 2022. Additionally, the ML phylogenetic tree showed the presence of six clusters: B.1.258 and sub-lineages, B.1.177, Alpha (B.1.1.7) and sub-lineages, Gamma (P.1.1), Delta (AY) and sub-lineages, Omicron (BA.1) and sub-lineages (Figure 5).

Interestingly, the viral strains from the married couple AM-1149 and AM-1184 (light blue dots) did not cluster with a significant bootstrap support. The B.1.177 isolates displayed different aa substitution on nsp2, PLpro, nsp7, nsp8, nsp13, and nsp15 regions (Supplementary Table S1). On the contrary, the viral strains isolated from the married couple AM-0607 and AM-0645 (dark blue dots) clustered with a good bootstrap value (bootstrap = 85). Both these B.1.258 isolates carried the same substitutions on whole genome, except for mutations I1192T on PLpro protein and N603D on S protein (Supplementary Table S1).

During the pandemic wave from January to March 2022, three subjects previously infected by B.1.177 (light blue dot: AM-0660a), Alpha (orange dot: AM-1281a), and Gamma (dark green dot: AM-0299a) acquired reinfection by Omicron or its sub-lineages (red dots: AM-0660b, AM-1281b and AM-0299b, respectively) (Figure 5).

### 3.5. Sanger Sequencing of Partial Spike Coding Region

The results obtained by sequencing the long amplicon of the spike protein are presented in Table 2. Fifty-seven partial sequences of the spike coding region yielded comparable results to whole genome sequencing. Specifically, in 2020, clade 20E (EU1) was detected in one sample, while in 2021, clades 20A, 20E, 20I (Alpha), and 20J (Gamma) were found in 19 samples. In 2022, all 37 samples belonged to the Omicron variant (clade 21K: BA.1, BA.1.1, BA.2).

## 4. Discussion

Rapid identification and characterization of emerging variants can inform public health strategies, such as implementing targeted interventions and adapting vaccination efforts to address the changing viral epidemiology [5,6]. The present study aimed to comprehensively investigate the characteristics of SARS-CoV-2 variants circulating among military and civilian personnel at an Air Force airport in Italy during three pandemic waves, spanning from November 2020 to March 2022. Military bases may be a hotspot for virus transmission and monitoring outbreaks is important to prevent spread to the general population. By analyzing a set of viral samples collected from the study population, we employed NGS and Sanger sequencing techniques to identify and to analyze various SARS-CoV-2 variants.

The results of the sequencing analysis revealed the presence of multiple SARS-CoV-2 variants circulating within the study population. Among several variants, the B.1.1.7 (Alpha, 9/67), the P.1 (Gamma, 4/67), and the B.1.617.2 (Delta, 4/67) VOCs were identified in 2020 and 2021. All these VOCs were previously characterized by the reduced susceptibility (potential immune escape) to antibodies generated during natural infection or after vaccination, as well as to mAbs therapies [9]. Additionally, each new VOC increased its transmissibility compared to the previous one, enhancing the number of new cases in the epidemiological landscape [9]. In December 2020, we detected B.1.177 (20E) and the B.1.1. B.1.258, B1.1.177, and Alpha were observed from January to February 2021. Between February and May 2021, Alpha was the major prevalent VOC in this community. In March 2021, we revealed the co-circulation of Alpha and Gamma VOCs. Delta VOC was detected from July 2021 to the end of the year. In 2022, only the Omicron (37/67) VOC, with its subvariants BA.1 (8/37), BA.1.1 (16/37), BA.1.1.1 (2/37), BA.1.15 (2/37), BA.1.17 (3/37), BA.1.18 (2/37), BA.1.8 (1/37), and BA.2 (3/37), were detected. Notably, BA.1 and BA.1.1 sub-lineages co-circulated during the first three months of 2022. Overall, this data agrees with the global epidemiology of SARS-CoV-2, since Omicron and its sub-lineages are extremely transmissible with a high infectivity rate, spreading much quicker than any other VOCs [9]. In the same time span, the Ministry of Health reduced public health restrictions in Italy, according to WHO guidelines [48]. Comparing our results with the overall Italian epidemiology, the appearance of VOCs and VOIs showed a similar trend at the military airport, suggesting that this community reflects the general population [12,49,50].

The genomic analysis of the 67 isolates highlighted a high number of aa changes in different regions of whole genomes. Starting from non-Omicron isolates, two major mutations characterize 3CLpro protein: G15S (2/30) and L232F (1/30). G15S was one of the most frequently identified missense mutations associated with 3cLpro inhibitor resistance, conferring reduced susceptibility to nirmatrelvir and ensitrelvir activity [21]. This mutation was fixed in some lineages, present in almost all strains in the lineages B.1.1.1, B.1.1.369, B.1.1.372, B.1.1.375, and lineage C, including the Lambda VOI [21]. Eighteen mutations were observed in the RdRp protein of non-Omicron VOC isolates. Some of the substitutions in nsp12 (polymerase) predict positive or negative functional effects. For example, Q822H predicts increased stability of the loop in the thumb domain [51]. The mutation at P227L influenced the tertiary Structure of nsp12, decreasing molecular flexibility on the protein structure. This substitution modifies the side chain, resulting in the alteration of intramolecular bonds in the pocket, which could lead to instability in co-factor binding (nsp7 and nsp8), ultimately modifying the proofreading complex and losing the structural integrity provided by proline [52]. Interestingly, the P323L substitution, emerging together with D614G, was found in all non-Omicron and Omicron new isolates. Very recently, structural analysis of P323L, G671S, and F694Y mutational patterns showed susceptibility to remdesivir, supporting the continued clinical use of this drug [53]. In the S glycoprotein of non-Omicron isolates, we found 12 unusual mutations, which were related to both B.1.258 and B.1.177 variants. Among these, the ΔH69–V70 deletion, observed in ten isolates, has been associated with increased infectivity and decreased susceptibility to neutralizing antibodies [54,55]. Furthermore, it has been identified in variants associated with immune escape in immunocompromised individuals treated with convalescent plasma [56]. Different changes were found in the function of the S protein. The T1117I substitution (AM-1059, AM-1060) determined a more stable interaction with a ligand (nelfinavir drug), but no differences with other genomes on transmissibility, severity, immune response, nor vaccine effectiveness were predicted [57,58]. Two isolates (AM-0424, AM-0882) carried the A262S mutation, which has demonstrated a slight increase of infectivity and decreased reactivities to mAb [59]. The P272L mutation carried by AM-0771, AM-0424, AM-0852, and AM-0882 allows evasion of T-cell responses in convalescent patients, and escape recognition by killer T-cells in vaccinated individuals [60,61]. Six unusual mutations were observed in Alpha isolates, including G1124V mutation, that appears to reduce epitope binding affinity of Human Leukocyte Antigen (HLA) alleles to a level that some are no longer able to bind, leading to virus escape from immune recognition [62]. Additionally, a total of 21 unusual mutations were observed in Gamma and Delta isolates, among which were L5F (AM-1988), L18F (AM-1059, AM-1060), and H655Y (AM-0272, AM-0299_a, AM-1002, AM-0120). The L5F mutation is in sites recognized by HLA, and it is seen to increase the epitope binding affinity for different HLA alleles [63]. Thus, the L5F is disadvantageous for the SARS-CoV-2 virus because the mutated epitope could enhance CD8 T cell recognition and killing through this improved interaction [63]. Additionally, it was the only unusual substitution to appear in BA.2 and BA.5 sub-lineages among 272 positive samples isolated in Southern Italy [64]. The L18F substitution is of significance because it has been found to compromise binding of neutralizing antibodies, allowing a much faster propagation in the presence of plasma antibodies collected from donors infected in the previous wave of the epidemic [65]. H655Y is responsible for alteration of entry properties and fusogenicity, using the preferential entry pathway without affecting the S cleavage status, and leading to modulations of tissue and cell tropism, and reduced pathogenicity [66]. V1176F (AM-0299a, AM-1002, AM-0120) is a recurrent S substitution that is frequently acquired by SARS-CoV-2 variants to increase viral fitness, improving interaction with heptad repeat 1 and enhance virus entry [67]. Three significant mutations in RBD (K417T/N, E484K and N501Y) are responsible for the escape from neutralizing mAbs and they could escape from both vaccine-induced sera and natural sera [68]. In particular, the glutamate to lysine substitution at position 484 (E484K) in the RBD of the spike protein is a single point mutation that affects binding by neutralizing antibodies, reducing the neutralizing activity of human polyclonal sera and thereby potentially rendering vaccine-induced immunity less protective [69]. The N501Y substitution also improved the affinity of the viral spike protein for cellular receptors, determining enhanced infection of the upper airway and viral transmission [70]. Other mutations that have been shown to potentially confer resistance to neutralization by mAbs are T20N, only detected in the Gamma P.1 VOC [71], and D138Y, which has higher binding affinity for human ACE2; as such, it could contribute to reduced neutralization by some mAbs, convalescent plasma, and sera from vaccines [72]. The G142D mutation (AM-1988, AM-B) at amino acid position 142 in the NTD of the spike protein was observed at multiple time points and across Delta VOC sub-lineages. This mutation is associated with higher viral load, further enhanced in the presence of another NTD mutation (T95I). The G142D mutation alters the surface topography of the NTD, more specifically, it disturbs the ‘super site’ epitope that binds NTD-directed neutralizing antibodies (nAbs) [73]. The T95I mutation occurs in the Delta variant and helps increase SARS-CoV-2 virulence [74]. T95I is a replacement of neutral and polar threonine by an acidic polar aspartic acid, enhancing viral load and adaptation by inducing the transition of a β-strand to an α-helix [74]. The same three isolates that showed the T95I mutation (AM-1894, AM-1988, AM-B) also carried three other amino acid changes: T19R, E156G/Δ157–158. T19R mutation was detected in the N terminal domain of the S protein and this mutation is plausible for increased infectivity, elevated transmissibility, and reduced sensitivity to anti-NTD neutralizing antibodies [75]. The NTD-specific E156G/Δ157–158 are, respectively, a change of glutamic acid to glycine at position 156 and a loss of two amino acids at positions 157 and 158. These mutations conferred an infectivity advantage, allowed immune escape, and reduced sensitivity to vaccine-induced antibodies [76]. Three isolates (AM-1988, AM-B, AM-1969) possessed two unique amino acid substitutions in their spike protein: S-P681R and S-D950N. P681R plays a key role in the Alpha-to-Delta variant replacement. This mutation is located at a furin cleavage site that separates the spike 1 (S1) and S2 subunits and it enhances the cleavage of the full-length spike to S1 and S2, which could improve cell-surface-mediated virus entry, increasing Delta-variant replication [77]. The D950N is a missense mutation that slightly promoted membrane fusion. It is located in the heptad repeat 1 (HR1) of the S2 subunit and is essential for the conformational change for virus fusogenicity [78]. A sub-lineage of the Delta variant, AY.4.2, exhibits distinguishing spike mutations Y145H (AM-B isolate) and A222V (found in nine isolates) that lie within the NTD of the spike. Through modelling, the Y145H substitution seems to decrease spike stability, but this has not been experimentally demonstrated [79].

On the other hand, a higher number of aa substitutions was observed in Omicron isolates, including 19 mutations such as Q173K (AM-0299_b) and A701V (AM-2094) already present in the Delta VOC. The Q173K resides within the N-terminal S1A that exhibits low conservation, which helps SARS-CoV-2 adapt to host cells and host immunity. This domain is bound very tightly by antibodies isolated from COVID-19 convalescent patients and conversion from a neutral to charged amino acid have the potential to alter interaction for antibody recognition [80]. A701V is near the furin cleavage site and seems to affect viral transmission, raising the affinity of protein interactions by enhancing the spike protein’s binding affinity and impacting antibody neutralization [74]. Three isolates belonging to BA.2 sub-lineage (AM-2110, AM-2119, AM-0302) carried the T19I, G142D, S371F, T376A, D405N, and R408S mutations. T19I in NTD spike was found to be significantly associated with Omicron mortality [81]. It caused significant evasion from NTD-targeted neutralizing mAbs [82], as well as R408S mutation that may reduce the efficacy of many antibodies [83]. G142D was located on the N-terminal domain of the spike protein and is involved in host cell attachment through diverse polysaccharide moieties. This mutation is known to be involved in increasing viral pathogenicity, vaccine breakthrough, and showed resistance to the mAbs [84]. S371F, in the ACE2-receptor-binding domain as well as the adjacent BA.2 specific T376A change, is reported to bear a significant fitness cost and impaired S infectivity, whilst also reducing processing efficiency and/or incorporation into viral pseudo particles [85]. The D405N mutation showed a dramatic loss of function by inhibiting spike/integrins interactions and consequently the virus ability to infect human lung microvascular endothelial cells, thus providing protection against virus-induced endothelial cell dysfunction [86].

Analysis of the N protein of non-Omicron isolates showed the presence of 12 unusual mutations among the non-Omicron VOC isolates, of which the A220V mutation, carried by seven B.1.177 variant isolates and one Alpha variant, stabilized the mutated N protein’s linker region, affecting RNA binding affinity [87], and L139F (AM-1601, AM-1602) localized in NTD of N protein that is most likely to affect the structure and stability of the N protein [88]. Eleven Alpha isolates also carried the double amino acid change (R203K and G204R plus S235F) in 6/9 isolates. The R203K/G204R are co-occurring mutations in the N protein responsible for the high transmissibility of lineages B.1.1.7 (Alpha) and P.1 (Gamma) due to their conferment of a replication advantage over preceding variants and increase virus fitness and virulence [89]. The S235F was seen to alter virus epitopes, causing changes in the specificity of certain antibodies, and altering vaccine-induced protection [90]. One B.1.258 isolate (AM-1059) showed the M234I mutation located in the link region between the RNA-binding domain and the dimerization domain of the N protein [91]. In particular, the S180I and A220V substitutions detected in two SARS-CoV-2 isolates, did not affect the negative results by the first-line rapid antigen test used for screening, targeting the N protein. Indeed, these substitutions were also carried by several isolates. The performance of this specific antigenic test is related to different features as already reported [27]. Among Omicron isolates, BA.1.1 sub-lineage showed uncommon mutations, such as K38I and T334A, while all BA.2 isolates showed the mutation S413R (serine→arginine) residing in the C-terminal end with no apparent effect on dimerization [92].

As discussed above, several known, unusual, and new mutations were studied by in vitro and/or in silico experiments to better define their function on pathogenicity, transmissibility, and disease severity of SAR-CoV-2 [53,57,66,73]. However, among the new isolates, we identified aa changes with a very low prevalence worldwide, carried on key regions, such as nsp3 (PLpro), nsp5 (3CLpro), nsp12 (RdRp), and ORF8 by non-Omicron and Omicron variants. In particular, we detected A1803V, D1809N, and A949T on PLpro and D216N on 3CLpro and P323L on RdRp Omicron coding regions, which are targets for new antiviral drugs. Very recently, a retrospective study regarding Delta VOC in Italy identified the aa changes with a positive selected pressure along the whole genome in order to evaluate the recurrent mutations for potential implications in the SARS-CoV-2 evolution. Comparing the newly generated sequences, we found several aa substitutions fixed in the viral population, offering a selective advantage: A318V and G339S (nsp2); P1228L (PLpro); L37F (nsp6); P323L (RdRp); P77L (nsp13); R289H (nsp14); L5F, T95I, G142D, N501Y, D614G, P681R, and D950N (S) [93]. In this paper, the G142D, L452R, D614G, P681R, and D950N substitutions (S region) and the P323L (RdRp region) were detected with higher frequency, identifying these substitutions as favoring viral adaptation [93]. In the Omicron era, it is important to monitor each new and known mutation already carried by previous VOCs, potentially involved in treatment, and in viral fitness.

To better characterize the positive cohort, we explored the demographic and referred clinical characteristics of individuals infected with different SARS-CoV-2 variants. The most infected people were male, 75.9% by non-Omicron variants and 62.2% by Omicron and sub-lineages, both group older than 50 years (100.0% versus 78.6%). Male and female subjects were not hospitalized even though 19/67 referred chronic diseases, therefore vaccines were certainly successful in preventing critical illness [94]. Several studies have reported a higher risk of mortality or hospitalization in male than female COVID-19 patients [9]. Non-specific symptoms of infection were reported more by people infected with Omicron and its sub-lineages (29/39) than individuals infected (10/39) in previous waves. COVID-19 symptoms and disease severity are directly related to interaction between SARS-CoV-2 variants and the human genome [95]. A total of 34/67 subjects had a previous diagnosis of COVID-19 or SARS-CoV-2 positivity, suggesting a second infection in the non-Omicron (12/67) time span, as well as in the Omicron era (22/67). The ML phylogenetic analysis clearly identified reinfections in people with available sequences during both infections. Three subjects previously infected by B.1.177 (AM-0660a), Alpha (AM-1281a) and Gamma (AM-0299a) acquired reinfection by Omicron or its sub-lineages (AM-0660b, AM-1281b and AM-0299b). Reinfection also occurred in people with two or three doses of vaccine. Alpha and Gamma VOCs manifested relatively mild escape from vaccination, while Omicron VOC showed a decrease in vaccine protection [20].

Phylogenetic analysis identified six clusters, with a bootstrap support >70, according to SARS-CoV-2 variants, such as B.1.258 and sub-lineages, B.1.177, Alpha and sub-lineages, Gamma, Delta and sub-lineages, Omicron and sub-lineages. Among these clusters, viral isolates from the couple AM-1149 and AM-1184 clustered with a low bootstrap support, suggesting two independent routes of transmission. On the other hand, the viral strains isolated from the couple AM-0607 and AM-0645 clustered with a bootstrap value of 85. Even if viral isolates were closely related, one of the two carried the additional I1192T and N603D substitutions on PLpro and S proteins, respectively, highlighting a continuous variability of the virus.

This study has some limitations. Symptoms and previous exposure to infection were noted from a questionnaire. The description and stratification of the cases based on the severity of symptoms was not applied due to the self-reporting of symptoms. Vaccination status was not available for all participants. From May 2021 to March 2022 only positive antigenic tests were confirmed by real-time RT-PCR. Finally, 57/67 samples were characterized using both NGS and Sanger methods due to unavailability of 10 viral RNA.

## 5. Conclusions

We provided valuable insights into the characteristics of SARS-CoV-2 variants circulating among military and civilian personnel at an Air Force airport as an important hotspot for viral spread. Our results indicated that partial sequencing of the spike coding region could yield comparable outcomes to whole genome sequencing, presenting itself as a rapid and cost-effective approach for identifying variants. In conditions of limited resources or when a result is urgently needed, a sequencing approach targeting a portion of the spike coding region can be used as an alternative to whole genome sequencing. However, while long PCR targeting the S gene may offer a quicker and targeted approach, it may miss important known, new, or unusual mutations in other genomic regions. These mutations could impact the virus spread, such as V1176F and T19R, the potential immune escape (i.e., well known E484K), the antiviral resistance, such as G15S, or the greater viral adaptation (P323L). It is noteworthy that the A1803V, D1809N, A949T, and the D216N substitutions (not yet characterized) were detected in target regions for new antiviral drugs. Finally, screening of the N region revealed the presence of S180I and A220V, which does not result in diagnostic failure by antigenic tests. The study’s findings highlight the importance of continuous genomic surveillance to monitor the evolution and spread of SARS-CoV-2 variants in the general population, as well as in restricted communities.

## Figures and Tables

**Table 1:** Demographic data, clinical manifestation, and contact tracing of sixty-seven positive people.

SARS-CoV-2 Variant	Non-Omicron			Omicron and Sub-Lineages		
Total Cases (n)	30			37		
Gender	Male	Female	Male (%)	Male	Female	Male (%)
	22	7	75.9	23	14	62.2
**Age (years old)**						
10–19	2	2	50.0	0	3	0.0
20–29	3	0	100.0	4	2	66.7
30–39	2	2	50.0	2	1	66.7
40–49	8	3	72.7	5	4	55.6
50–59	6	0	100.0	11	3	78.6
60–69	0	0	-	0	1	0.0
≥70	0	0	-	1	0	100.0
not available	1					
**Clinical manifestations**						
Non-specific symptoms of infection	7	3	70.0	17	12	58.6
COVID-19 symptoms	12	5	70.6	18	13	58.1
Previous COVID-19 diagnosis	6	0	100.0	4	7	36.4
Previous SARS-CoV-2 positive swab	5	1	83.3	4	7	36.4
Chronic diseases	3	0	100.0	7	8	46.7
**Contact tracing**						
Contact with COVID-19 sick people in the previous 14 days	9	4	69.2	13	12	52.0
Contact with positive people to the SARS-CoV-2 swab in the previous 14 days	11	4	73.3	14	13	51.9
not available	1					

**Table 2:** Variant and clade classification using Sanger sequencing of the spike region.

Sampling Period		ID Sample	Variant/Clade
2020	3 December	AM-70	20E (EU1)
2021	22 January	AM-607	20A
	26 January	AM-660	20E (EU1)
	02 February	AM-771	20E (EU1)
	05 February	AM-424	20E (EU1)
	09 February	AM-882	20E (EU1)
	16 February	AM-1059	20A
	16 February	AM-1060	20A
	18 February	AM-1095	20E (EU1)
	24 February	AM-1148	20I (Alpha, V1)
	26 February	AM-1184	20E (EU1)
	05 March	AM-1309	20I (Alpha, V1)
	16 March	AM-272	20J (Gamma, V3)
	19 March	AM-1397	20I (Alpha, V1)
	23 March	AM-299	20J (Gamma, V3)
	30 March	AM-433	20I (Alpha, V1)
	30 March	AM-1281	20I (Alpha, V1)
	22 April	AM-1601	20I (Alpha, V1)
	22 April	AM-1602	20I (Alpha, V1)
	13 December	AM-B	21J (Delta)
2022	10 January	AM-2037	Omicron BA.1.1
	10 January	AM-2038	Omicron BA.1.1
	10 January	AM-2039	Omicron BA.1.1
	10 January	AM-2040	Omicron BA.1.1
	10 January	AM-2045	Omicron BA.1.1
	10 January	AM-2046	Omicron BA.1.1
	10 January	AM-2050	Omicron BA.1
	17 January	AM-1739	Omicron BA.1
	17 January	AM-1741	Omicron BA.1
	17 January	AM-2058	Omicron BA.1
	17 January	AM-2059	Omicron BA.1
	17 January	AM-2060	Omicron BA.1
	17 January	AM-2071	Omicron BA.1.1
	17 January	AM-2072	Omicron BA.1
	20 January	AM-2092	Omicron
	20 January	AM-2094	Omicron
	24 January	AM-2096	Omicron
	24 January	AM-2097	Omicron
	27 January	AM-1273	Omicron
	27 January	AM-1281	Omicron
	10 February	AM-2074	Omicron
	10 February	AM-2109	Omicron
	10 February	AM-2110	Omicron
	10 February	AM-2111	Omicron
	10 February	AM-2116	Omicron
	10 February	AM-2117	Omicron
	10 February	AM-2118	Omicron
	10 February	AM-2119	Omicron
	10 February	AM-2120	Omicron
	24 February	AM-1438	Omicron
	14 March	AM-0236	Omicron
	14 March	AM-0299/B	Omicron
	14 March	AM-0302	Omicron
	14 March	AM-0660	Omicron
	14 March	AM-2122	Omicron
	14 March	AM-2123	Omicron
	14 March	AM-2124	Omicron

## Data Availability

All sequences can be retrieved from GenBank under accession numbers: OR761892–OR761958.

## Supplementary Materials

The following supporting information can be downloaded at: https://www.mdpi.com/article/10.3390/microorganisms11112711/s1, Table S1: Major amino acidic changes along the proteins of non-Omicron VOCs isolates; Table S2: Major amino acidic changes along the proteins of Omicron VOC and its sub-lineage isolates.
